# Pharmacological Treatments in Alcohol Use Disorder and Risk of Alcohol‐Related Hospitalizations: A Register Study

**DOI:** 10.1111/acps.13802

**Published:** 2025-03-17

**Authors:** Patrick Bach, Johan Franck, Jonas Hällgren, Härje Widing, Mika Gissler, Jeanette Westman

**Affiliations:** ^1^ Department of Clinical Neuroscience Karolinska Institutet Stockholm Sweden; ^2^ Department of Addictive Behavior and Addiction Medicine Central Institute of Mental Health, University of Heidelberg, Medical Faculty Mannheim Mannheim Germany; ^3^ Department of Data and Analytics Finnish Institute for Health and Welfare Helsinki Finland; ^4^ Academic Primary Care Centre. Region Stockholm Stockholm Sweden; ^5^ Department of Molecular Medicine and Surgery Karolinska Institutet Stockholm Sweden; ^6^ Department of Child Psychiatry Turku University Hospital, Turku University Turku Finland; ^7^ Department of Neurobiology, Care Sciences and Society Karolinska Institutet Stockholm Sweden; ^8^ Department of Health Care Sciences Marie Cederschiöld University Stockholm Sweden

**Keywords:** acamprosate, alcohol use disorder, disulfiram, hospitalization, medication, naltrexone

## Abstract

**Objectives:**

Despite the high prevalence of alcohol use disorder (AUD), only a minority of patients receive recommended pharmacological treatments, possibly owing to uncertainty about the real‐world effectiveness of these medications. Here, we analyzed nationwide, register‐based data to investigate the association between approved AUD medications (naltrexone, acamprosate, disulfiram, and nalmefene) and the risk of alcohol‐related hospitalizations among individuals with AUD.

**Methods:**

People aged 18–64 with a registered first‐time diagnosis of AUD between 2009 and 2019 (*N* = 93,727) were identified from the Swedish National Patient Register. Cox regression models were used to analyze the association between AUD medication exposure and the risk of alcohol‐related hospitalizations.

**Results:**

Exposure to naltrexone (hazard ratio [HR] = 0.80; 95% confidence interval [CI] = 0.73**–**0.87) or disulfiram (HR = 0.83, 95% CI = 0.79**–**0.88) as monotherapy, or a combination of naltrexone/disulfiram (HR = 0.68, 95% CI = 0.49**–**0.96), or disulfiram/acamprosate (HR = 0.57 95% CI = 0.44**–**0.74) was significantly associated with a lower risk of alcohol‐related hospitalizations compared to periods without exposure to any of these medications. In contrast, no significant associations were observed for acamprosate, nalmefene, or the combination of acamprosate/naltrexone. Sensitivity analyses in individuals with severe AUD and stratified subgroup analyses by different socioeconomic groups confirmed the robustness of the results.

**Conclusion:**

Results indicate a significant association between disulfiram and naltrexone monotherapy, as well as the combination of disulfiram with naltrexone or acamprosate, with a lower risk of alcohol‐related hospitalizations among individuals with AUD. Low prescription rates suggest that AUD medications are currently underutilized. Increasing the availability of these medications for individuals with AUD could help reduce alcohol‐related hospitalizations.

**Summary**

- Significant outcomes
  ○ Exposure to pharmacological treatments for alcohol use disorder (naltrexone, disulfiram, acamprosate, nalmefene) is associated with a lower risk of alcohol‐related hospitalizations in individuals with alcohol use disorder.
  ○ Exposure to naltrexone or disulfiram, or a combination of both, was significantly associated with a lower risk of alcohol‐related hospitalizations, while acamprosate and nalmefene were not.
  ○ Pharmacological treatments are currently underutilized among individuals with alcohol use disorder.
- Limitations
  ○ The estimation of exposure periods relied on prescription data.
  ○ Only individuals who had contact with the healthcare system and received an AUD diagnosis were considered in the analyses.

## 1 Introduction

Alcohol use disorder (AUD) is one of the most prevalent and devastating psychiatric disorders worldwide and a major risk factor for disability and death [1]. Several medications have been approved to treat AUD by the European Medicines Agency (EMA) and the Swedish Medical Products Agency, including naltrexone (NTX), acamprosate (ACAM), nalmefene (NMF), and disulfiram (DIS). The Swedish National Board of Health and Welfare [2] and international guidelines [3, 4, 5] recommend offering these medications to all individuals with severe AUD (i.e., alcohol dependence). Randomized controlled trials support the efficacy of these medications [6, 7, 8, 9] in preventing relapse or reducing alcohol use, but only a minority of individuals with AUD currently receive a prescription for these medications. A study using Swedish register data also reported a lower hospitalization risk in individuals with AUD when treated with NTX as monotherapy or in combination with ACAM or DIS [10]. However, studies also indicate substantial variability in patients' responses to pharmacological treatment [11], raising questions about the real‐world efficacy of these medications. Uncertainty about the efficacy and potential harm of AUD medication in treatment practice may contribute to low prescription rates in Sweden [12], other EU countries [13, 14] and the United States [15], which indicate that only about 3%–25% of individuals with AUD receive pharmacological AUD treatment.

## 2 Aims of the Study

The primary aim was to examine the association between AUD medication exposure and the risk of alcohol‐related hospitalization among individuals with AUD. The secondary aims were to investigate the effects of sociodemographic factors and comorbidities on the observed associations and to determine prescription rates of different AUD medications.

## 3 Methods

We conducted an analysis of Swedish nation‐wide register data. The data were retrieved from nation‐wide registers, which comprise the entire population registered in Sweden. Patient data from inpatient and specialized outpatient care was retrieved from the National Patient Register [16] and prescription data from the Prescribed Drug Register [17]. Dates of death were gathered from the Causes of Death Register [18] and demographic data from the Longitudinal Integration Database for Health Insurance and Labor Market Studies (LISA) Register. All Swedish citizens and residents are assigned a unique personal identification number, which enables linkage between different registers.

The study was approved by the Swedish Ethical Review Authority (decision 2019–00516). No informed consent was required due to the anonymity of the register data.

### 3.1 Study Population

From the total population, we identified individuals aged 18–64 years with a first‐time diagnosis of an alcohol‐related disorder during the observational period from 2009 to 2019 in inpatient or outpatient care. The AUD diagnosis was defined according to the 10th revision of the International Classification of Diseases and Related Health Problems (ICD‐10) (ICD‐10: F10.0‐F10.9). Individuals diagnosed with an alcohol‐related disorder before 2009 (i.e., between 1997 and 2008), or who had received a prescription for an AUD medication prior to the diagnosis of an AUD were excluded from the cohort. The total cohort included *N* = 93,727 individuals with AUD. These individuals were followed in the register, starting from the date of the first diagnosis of an alcohol‐related disorder during the period between 2009 and 2019 and ending at the age of 65 years, death, date of emigration, or end‐of‐study follow‐up (i.e., December 31, 2019), whichever occurred first.

### 3.2 Exposure to Medication

Data on prescription of AUD medication were retrieved from the Prescribed Drug Register [17], including the prescription date, the anatomical therapeutic chemical (ATC) classification code, the defined daily dose (DDD), and information on drug packaging and formulation for DIS (ATC N07BB01), ACAM (N07BB03), NTX (N07BB04), and NMF (N07BB05). We estimated exposure periods from the retrieved data, setting the start of the exposure period to the date of initial prescription, with duration and end of the exposure period estimated from purchased DDDs of the respective medication (i.e., prescription date + DDDs = end date of exposure period).

To investigate the effects of combined treatment with multiple AUD medications, the exposure periods for combined treatments with two or more AUD medications during the observational period were calculated similarly. Co‐prescription was defined as the prescription of two AUD medications on the same date, with exposure beginning on the co‐prescription date and ending on the date of the shortest DDD of the two medications. All other combinations of more than two AUD medications, combinations of any AUD medication with NMF, and cases where a second prescription was issued during the exposure period for another medication (i.e., delayed, but overlapping exposure periods) were combined into a “mixed” treatment group.

### 3.3 Outcome Measures

The primary outcome measure was inpatient hospitalizations due to alcohol‐related problems (main diagnosis F10.0‐F10.9). Hospitalizations with discharge and admission on the same date were considered to be the one event, due to the absence of relevant exposure periods between both events. In addition, we conducted descriptive analyses of prescription rates across different socioeconomic groups and individuals with and without somatic or psychiatric comorbidities.

### 3.4 Statistical Analyses

Hospitalizations were treated as recurrent events and analyzed using a between‐individual cox regression model [19] with time since the last event (i.e., alcohol‐related hospitalization) as the underlying time scale. This model assumes that all observed event times are independent, irrespective of whether these event times attribute to the same patient or to different patients. Hazard ratios with corresponding 95% confidence intervals (CI) were calculated using robust standard errors [20] to correct for possible dependence between hospitalizations within individuals. To ensure the suitability of the Cox regression model, the proportional hazards assumption was checked by visual inspection of the scaled Schoenfeld residuals versus time, with a smoothed curve superimposed to assess any trends. The analyses were adjusted for time‐invariant covariates, including sex and education level at baseline, as well as time‐varying covariates, including age, number of previous exposure periods to AUD medication (0, 1, 2, ≥ 3), number of previous alcohol‐related (F10.0‐F10.9) hospitalizations (0, 1, 2, ≥ 3), cohabitation status, comorbid substance use disorders (F11‐F19), severity of somatic comorbidities (Somatic comorbidity index, for details see Table S1) and benzodiazepine exposure (calculated as one DDD per day, similarly to the exposure periods for AUD medication). Covariates were selected considering prior research that indicated potential influences of these covariates on either the likelihood of receiving AUD medication prescriptions [12] or the likelihood of getting hospitalized due to AUD [10] and to account for sociodemographic disparities in treatment access and health outcomes, as well as influences of comorbidities and concomitant treatments and disease severity, and prior treatment attempts.

### 3.5 Sensitivity Analyses

Sensitivity analyses included only those individuals with a first‐time diagnosis of alcohol dependence (ICD‐10 code: F10.2), that is, a severe AUD. This approach was chosen to replicate analyses in the population for which treatment guidelines recommend treatment with AUD medications and who are the target group for these pharmacological interventions.

Additional stratified subgroup analyses for the main outcome were performed for both sexes (male/female), different age groups (18–24; 25–34; 35–44; 45–54; 55–64 years), education levels (unknown or no completed primary education/primary education/secondary education/university), cohabitation status (yes/no) and severity of somatic comorbidities (approximated using a modified Charlson Comorbidity Index [21, 22]: 0; 1–2; 3–5; ≥ 6, for details see Table S1) separately. This approach was chosen to allow estimation of effect sizes in these subgroups and assess the robustness of the results of the primary analysis.

To confirm the robustness of the between‐subject analyses, we also conducted additional within‐subject analyses using a stratified Cox regression model, in which each individual served as their own reference, to control for time‐invariant confounders. This approach accounts for time‐invariant individual characteristics by comparing periods of medication exposure to non‐exposure within the same individual, thereby controlling for confounding due to stable personal factors such as genetics, baseline health status, and socioeconomic background. In this model, only individuals that are exposed at least once and that have at least one hospitalization during the follow‐up period contribute to the model. The within‐individual analyses were adjusted for time‐varying covariates, including age, number of previous exposure periods to AUD medication, cohabitation status, comorbid substance use disorders, severity of somatic comorbidities, and benzodiazepine exposure.

Analyses were performed using SAS software (version 9.4, SAS Institute, Cary, North Carolina, US).

## 4 Results

The study cohort included a total of 93,727 individuals with a diagnosis of an alcohol‐related disorder. Of those, 57,730 (61.6%) were male, and the mean age was 37.0 ± 14.6 years. The median follow‐up time was 5.0 years. The clinical and socio‐demographic characteristics of the cohort are depicted in Table 1. During the follow‐up, 23,649 (25.2%) of the individuals were prescribed AUD medications at least once: 12113 (12.9%) received DIS, 8014 (8.6%) ACAM, 10319 (11.0%) NTX, 547 (0.6%) NMF.

**TABLE 1:** Description of the cohort (*N* = 93,727), including all residents aged 18–64 years, living in Sweden with registered first‐time treatment contact due to AUD during the years 2009–2019.

Variable	*N* (%)
Sex	
Female	35,997 (38.4)
Age	
Mean (standard deviation)	36.98 (14.6)
Education level at study entry	
University education	19,033 (20.3)
Secondary education	49,165 (52.5)
Primary education	23,811 (25.4)
Unknown/no primary education	1718 (1.8)
Cohabitation status during follow‐up	
Yes	43,964 (46.9)
Somatic comorbidity index during follow‐up	
≥ 6	4887 (5.2)
3–5	12,191 (13.0)
1–2	25,132 (26.8)
0	51,517 (55)
Benzodiazepine use during follow‐up	
Yes	19,127 (20.4)
Comorbid SUD (F11‐F19) at study entry	
Yes	10,134 (10.8)
Number of deaths during follow‐up	
Yes	4565 (4.9)
Diagnosis at study entry	
F10.9	3121 (3.3)
F10.8	328 (0.3)
F10.7	152 (0.2)
F10.6	176 (0.2)
F10.5	325 (0.3)
F10.4	406 (0.4)
F10.3	3974 (4.2)
F10.2	23,340 (24.9)
F10.1	17,086 (18.2)
F10.0	44,819 (47.8)
AUD‐related hospitalizations during follow‐up	
Mean (Standard Deviation)	0.74 (2.2)
Number of AUD‐related hospitalizations during follow‐up	
≥ 3	5408 (5.8)
2	4495 (4.8)
1	25,389 (27.1)
0	58,435 (62.3)
Number of exposure periods during follow‐up	
≥ 3	5400 (5.8)
2	5246 (5.6)
1	13,003 (13.9)
0	70,078 (74.8)
Prescription of any AUD medication during follow‐up	
Yes	23,649 (25.2)
Prescription of any AUD medication within first year after diagnosis	
Yes	15,941 (17.0)
Acamprosate	
Yes	8014 (8.6)
Disulfiram	
Yes	12,113 (12.9)
Nalmefene	
Yes	547 (0.6)
Naltrexone	
Yes	10,319 (11)

Prescription rates of AUD medication were unequally distributed across sociodemographic groups, with the lowest prescription rates seen in the youngest cohort (18–24 years) and among individuals with the lowest level of education (see Table 2).

**TABLE 2:** Depiction of the prescription rates of medication for alcohol use disorder across sociodemographic groups (rates were defined as the number of individuals in the population that received a prescription of any approved AUD medication during the first 12 months following the initial AUD‐diagnosis [F10.0‐F10.9] during the observational period from 2009 to 2019).

Prescription rates of AUD medication	Yes, *N* (%)
Sex	
Male	10,133 (17.6)
Females	5808 (16.1)
Age groups	
18–24	1393 (5)
25–34	2573 (14.7)
35–44	3765 (25)
45–54	4799 (27)
55–64	3411 (22.4)
Diagnostic categories	
F10.0	1840 (4.1)
F10.1	4362 (25.5)
F10.2	8032 (34.4)
F10.3	1080 (27.2)
F10.4	75 (18.5)
F10.5	50 (15.4)
F10.6	9 (5.1)
F10.7	4 (2.6)
F10.8	56 (17.1)
F10.9	433 (13.9)
Education levels	
Unknown/no primary education	175 (10.2)
Primary education	3123 (13.1)
Secondary education	8408 (17.1)
University education	4235 (22.3)
Somatic comorbidity index	
0	10,346 (16.5)
1–2	3511 (17)
3–5	1700 (20.4)
6–10	384 (17.8)
≥ 11	10,346 (16.5)
Cohabitation	
No	9649 (15.9)
Yes	6292 (18.9)

**FIGURE 1:**
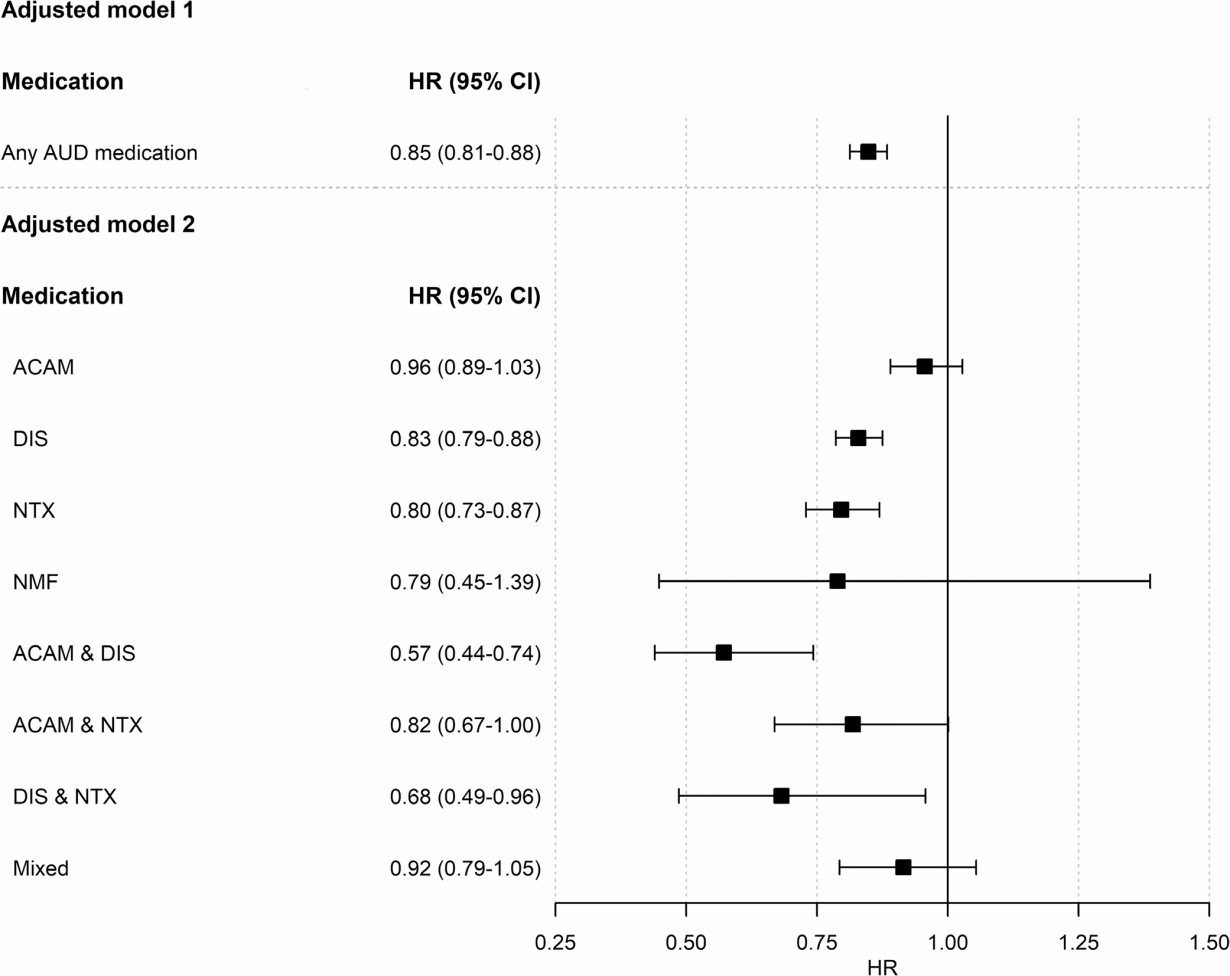
Adjusted hazard ratios (HRs) and 95% confidence intervals (CIs) for the risk of hospitalization due to alcohol use disorder (AUD) for the population of *N* = 93,727 individuals with AUD during pharmacotherapy compared with no use of medication. Models were adjusted for age, number of previous exposure periods to AUD medication (0, 1, 2, ≥ 3), number of previous alcohol‐related (ICD‐10 codes: F10.0‐F10.9) hospitalizations (0, 1, 2, ≥ 3), cohabitation status, comorbid substance use disorders (F11‐F19), severity of somatic comorbidities (SCI) and benzodiazepine exposure (ACAM = acamprosate, DIS = disulfiram, NMF = nalmefene, NTX = naltrexone, and mixed = use of more than two AUD medications or combination with nalmefene).

A total of 35,292 (37.7%) individuals had an alcohol‐related hospitalization during follow‐up. Results of the adjusted primary Cox regression model showed that exposure to any AUD medication (NTX, DIS, ACAM, or NMF) was associated with a significantly lower hospitalization risk during follow‐up when compared with periods without exposure to AUD medication (HR = 0.85, 95% CI = 0.81–0.88, *p* < 0.0001, see Figure 1 *—Adjusted model 1*).

When examining the association between exposure to monotherapy and combination therapy with specific AUD medications and hospitalization risk, we found that both NTX (HR = 0.80, 95% CI: 0.73–0.87, *p* < 0.0001) and DIS (HR = 0.83, 95% CI: 0.79–0.88, *p* < 0.0001) as monotherapies were significantly associated with a lower hospitalization risk. In contrast, no significant effects were observed for ACAM (HR = 0.96, 95% CI: 0.89–1.03, *p* = 0.2224) or NMF (HR = 0.79, 95% CI: 0.45–1.39, *p* = 0.4095).

Significant associations were also observed for the combination of NTX & DIS (HR = 0.68, 95% CI: 0.49–0.96, *p* = 0.0267) and DIS & ACAM (HR = 0.57, 95% CI: 0.44–0.74, *p* < 0.0001), whereas no significant effects were found for NTX & ACAM (HR = 0.82, 95% CI: 0.67–1.00, *p* = 0.0509) or for other combinations categorized under “mixed” medication (HR = 0.92, 95% CI: 0.79–1.05, *p* = 0.2186) (see Figure 1 *—Adjusted Model 2*).

### 4.1 Sensitivity Analyses

**FIGURE 2:**
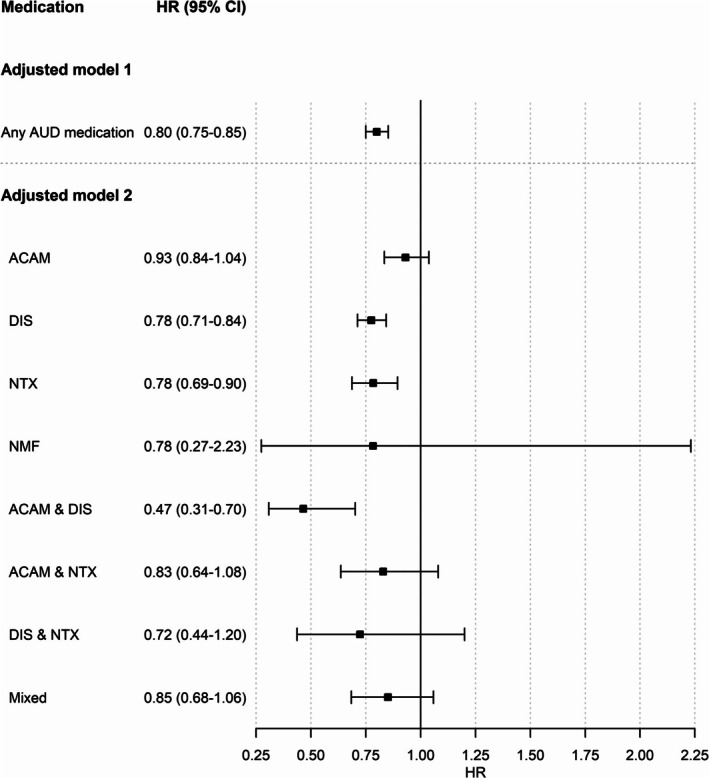
Adjusted hazard ratios (HRs) and 95% confidence intervals (CIs) for the risk of hospitalization due to alcohol use disorder (AUD) in the subpopulation of *N* = 23,340 individuals who were diagnosed with alcohol dependence (i.e., severe AUD) (ICD‐10 code: F10.2) at baseline. Models were adjusted for age, number of previous exposure periods to AUD medication (0, 1, 2, ≥ 3), number of previous alcohol‐related (F10.0‐F10.9) hospitalizations (0, 1, 2, ≥ 3), cohabitation status, comorbid substance use disorders (F11‐F19), severity of somatic comorbidities (SCI) and benzodiazepine exposure (ACAM = acamprosate, DIS = disulfiram, NMF = nalmefene, NTX = naltrexone, and mixed = use of more than two AUD medications or combination with nalmefene).

The results of the sensitivity analysis among individuals with a first‐time diagnosis of alcohol dependence during the study period (ICD‐10: F10.2, *n* = 23,340) corroborated the significant association between the exposure to any AUD medication, to NTX or DIS alone, and to the combination of DIS & ACAM with a significantly lower hospitalization risk, while other associations did not yield significance (see Figure 2).

**FIGURE 3:**
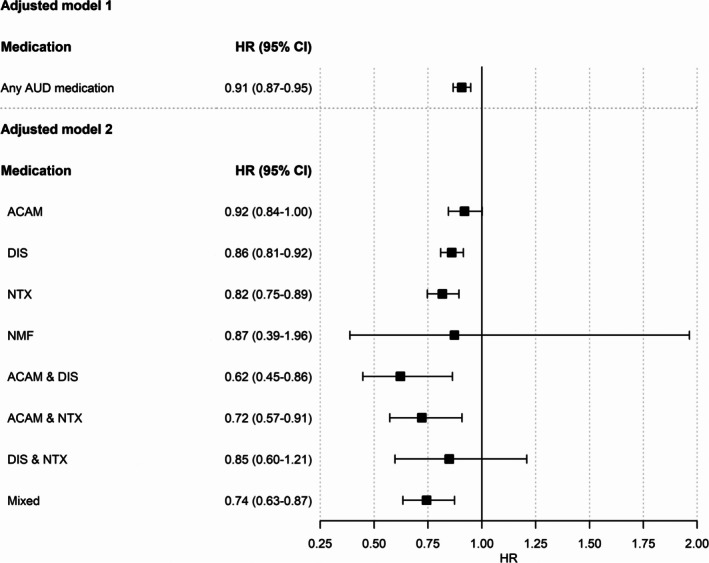
Results of the within‐subject sensitivity analysis investigating the association of exposure to any AUD medication (model 1) or specific AUD medications (model 2) with the risk of alcohol‐related hospitalizations among individuals who were exposed at least once and had at least one hospitalization during follow‐up (*n* = 8232). Adjusted hazard ratios (HRs) and 95% confidence intervals (CIs) for the risk of hospitalization due to alcohol use disorder (AUD) during pharmacotherapy compared with no use of medication are shown for the different medication groups. Models were adjusted for age, number of previous exposure periods to AUD medication (0, 1, 2, ≥ 3), cohabitation status, comorbid substance use disorders (F11‐F19), severity of somatic comorbidities (SCI) and benzodiazepine exposure (ACAM = acamprosate, DIS = disulfiram, NMF = nalmefene, NTX = naltrexone, and Mixed = use of more than two AUD medications or combination with nalmefene, * significant with *p* < 0.05).

Additional within‐individual analyses, including *n* = 8232 individuals, corroborated the findings of the primary analysis. Results confirmed the significant association between exposure to any AUD medication, to NTX or to DIS alone, and to the combination of DIS & ACAM with a significantly lower hospitalization risk, while the association between exposure to ACAM alone or to the combination of DIS & NTX and lower hospitalization risk did not yield significance (see Figure 3).

### 4.2 Subgroup Analyses

Stratified subgroup analyses demonstrated significant associations of exposure to AUD medication and the risk of alcohol‐related hospitalizations in both men (HR = 0.82, 95% CI = 0.78–0.86, *p* < 0.0001) and women (HR = 0.90, 95% CI = 0.84–0.97, *p* = 0.0032, see Figure S1). Different associations were observed across age groups. While a lower risk of AUD‐related hospitalizations during exposure to AUD medication was observed among individuals of 35 years and older (HR range = 0.74–0.85, *p* value range = 0.0001–0.0003, see Figure S1), no significant association was observed for the age group 25–34 years (HR = 1.03, 95% CI = 0.94–1.12, *p* = 0.5884) and a higher risk was observed for the youngest age group of 18–24 years (HR = 1.19, 95% CI = 1.03–1.39, *p* = 0.0201). For education status, we observed a significant association between exposure to AUD medication and lower hospitalization risk in individuals with primary or higher education (HR range = 0.82–0.90, *p* value range = 0.0001–0.0184, see Figure S1), while no significant association could be observed in the group with unknown education status or without completed primary education (HR = 0.82, 95% CI = 0.63–1.06, *p* = 0.1277). Concerning cohabitation status, we observed significant associations of AUD medication on hospitalization risk in individuals living in a partnership (HR = 0.79, 95% CI = 0.73–0.86, *p* < 0.0001) and in individuals living single (HR = 0.87, 95% CI = 0.82–0.91, *p* < 0.0001). We also found significant associations between exposure to AUD medication and hospitalization risk across all somatic comorbidity severity groups (HR range = 0.78–0.85, *p* value range = 0.0001–0.0021, see Figure S1).

## 5 Discussion

Exposure to AUD medications was significantly associated with a reduced risk of alcohol‐related hospitalizations. Specifically, NTX and DIS monotherapy, as well as the combination of NTX/DIS and ACAM/DIS, demonstrated significant associations with lower hospitalization risk. In contrast, ACAM and NMF monotherapy did not show significant associations.

These findings align with previous register‐based studies, which also reported lower hospitalization risks for NTX and DIS [10] and clinical trials, which reported significant effects of both medications on abstinence rates and relapse risk [7, 23, 24, 25]. However, while clinical trials support ACAM's role in reducing relapse rates [6, 23, 26], its association with hospitalization risk was not significant in the present and also previous register studies [10]. The lack of a significant association between ACAM and hospitalization risk may thus be due to a lack of efficacy in reducing heavy drinking episodes [6, 23, 26], which are closely linked to alcohol‐related hospitalizations (e.g., due to intoxication). The results for NMF, often prescribed for harm reduction rather than abstinence, were similarly non‐significant. These Results are in line with previous research on register data [10] and might be explained by the limited effectiveness of NMF and the low number of events in this group, resulting in wide confidence intervals and limited power to detect associations with hospitalization risk.

A notable finding was the greater effect of combined pharmacotherapy. The observed synergistic effects of NTX/DIS and ACAM/DIS may stem from complementary mechanisms: DIS's aversive properties combined with NTX's or ACAM's anti‐craving effects could enhance treatment adherence and effectiveness [27]. While clinical guidelines primarily recommend monotherapy, these findings suggest that specific medication combinations might enhance efficacy and warrant further investigation.

In line with prior research on European and U.S. prescription data [12, 13, 14, 15], we observed rather low prescriptions, particularly among younger individuals and those with lower education levels. This indicates that these medications are currently underutilized, despite evidence supporting their efficacy. This underscores the need for increased awareness and accessibility of pharmacological treatments for AUD.

### 5.1 Limitations

The conclusions on medication effectiveness that can be drawn from the presented data set are limited by the associative nature of register data and missing information on the severity of AUD. We tried to mitigate this by only considering cases with the first AUD diagnosis during the observational period, in order to exclude individuals with a long history of AUD. In addition, we conducted additional sensitivity analyses in individuals with diagnosed alcohol dependence (F10.2), to limit the population to individuals for which treatment with AUD medication is recommended by treatment guidelines. Furthermore, our analysis mitigated potential bias by different AUD treatment histories by including the number of previous exposure periods and the number of prior hospitalizations as covariates. It should, however, be noted that we could not investigate the potential effect of other covariates, such as family history of AUD or treatment motivation, which could have an impact on the response to AUD treatment. Further studies are needed to investigate the potential influence of such factors on treatment outcomes. There is a risk that the associations between exposure to AUD medication and hospitalization risk were underestimated, as the register only captures prescriptions collected at pharmacies and not those procured through other channels. Still, this would only lead to an underestimation of the number of individuals receiving AUD medication and of the association of AUD medication with hospitalization risk. In addition, the estimation of exposure periods assumed that individuals took their medication as prescribed, which most likely does not precisely reflect treatment reality. Discontinuation of medication intake by patients could have contributed to underestimating the associations of AUD medication and hospitalization risk. It should be noted that register data does not provide information on whether unexposed individuals declined pharmacological AUD treatment due to, e.g., a lack of motivation to remain abstinent. Treatment motivation could thus be different in individuals that were exposed versus unexposed, which could have impacted the observed risk of AUD‐related hospitalizations [28, 29, 30, 31]. Further studies are needed to better understand the interactions between pharmacological effects, motivational factors and treatment outcomes in AUD. Still, presented results are informative because they reflect real‐world treatment practice, for which it is important to understand whether handing out a prescription for a certain medication is associated with differences in the rates of clinical outcomes (e.g., hospitalizations), because adherence—in most settings—cannot be monitored perfectly. Furthermore, there was no information on concurrent non‐pharmacological AUD treatments (e.g., psychosocial therapy) during the observational period, which could have contributed to different associations with hospitalization risk, but an unequal distribution across medication groups is not to be expected when considering current treatment guidelines and treatment practice.

## 6 Conclusion

Presented results indicate that NTX and DIS are significantly associated with a reduced risk of alcohol‐related hospitalizations in individuals with AUD. Notably, combinations of AUD medications with different mechanisms of action, such as DIS/NTX and DIS/ACAM, show even stronger associations with reduced hospitalization risk. These findings highlight the protective effect of AUD medication, yet their overall impact remains limited by the observed low prescription rates. In line with treatment guidelines, our results underscore the recommendation for the prescription and utilization of pharmacological AUD treatments in individuals with AUD.

## Author Contributions

P.B., J.H., H.W., M.G., J.F., and J.W. were responsible for the study concept and design. J.H., H.W., P.B., M.G., and J.W. conducted the data analysis and interpretation of findings. P.B., J.W., and J.F. drafted the manuscript. All the authors critically revised the manuscript for important intellectual content. All the authors critically reviewed the content and approved the final version for publication.

## Ethics Statement

This is an observational study. The study was approved by the Swedish Ethical Review Authority (decision 2019–00516).

## Consent

The authors have nothing to report.

## Conflicts of Interest

The authors declare no conflicts of interest.

## Peer Review

The peer review history for this article is available at https://www.webofscience.com/api/gateway/wos/peer‐review/10.1111/acps.13802.

## Supporting information


Data S1.

## Data Availability

The data that support the findings of this study are available from the Swedish National Patient Register, the Swedish Prescribed Drug Register, the Swedish Causes of Death Register, and the Longitudinal Integration Database for Health Insurance and Labor Market Studies register (https://www.socialstyrelsen.se/en/statistics‐and‐data/registers/). Restrictions apply to the availability of these data, which were received by the authors upon request and after approval by the competent authorities. Data are available from these sources after submission of a scientific request and permission by the competent authorities.
