# Supplementary material for: Pharmacological Treatments in Alcohol Use Disorder and Risk of Alcohol‐Related Hospitalizations: A Register Study
Source: Acta Psychiatr Scand. 2025 Mar 17;152(2):94–103. doi: 10.1111/acps.13802 (PMC12213011; doi:10.1111/acps.13802)

# Supplements

## Supplementary Tables

## Supplementary Table S1. Depiction of the ICD-10 codes and respective weights, which were considered when computing the modified Somatic Comorbidity Index (CCI), derived from ([19](#_ENREF_19)). The total score of the CCI was calculated by summing up the weights of the respective ICD-10 diagnoses of an individual. Due to the fact that ICD-10 diagnosis data was in some instances only available for an entire diagnostic category/ICD-10 trunk, all diagnoses that fell in these ICD-10 trunks were considered and given the same weight. It should be noted that this approach might have over-estimated the true severity of comorbid diseases, because any disease within the ICD-10 trunk was counted (AIDS = acquired immunodeficiency syndrome, HIV = Human Immunodeficiency Virus).

| **Name** | **ICD-10** | **Assigned Weight** |
| --- | --- | --- |
| AIDS/HIV | B20, B21, B22, B23, B24, F024, O94-O99, R70-R79, Z00-Z13, Z20-Z29, Z70-Z76 | 6 |
| Cerebrovascular disease | G40-G47, I60-I69 | 1 |
| Chronic heart failure | I30-I52 | 1 |
| Chronic obstructive pulmonary disease | J40-J47 | 1 |
| Dementia | F00, F01, F02, F03, F051, G30-G32 | 1 |
| Disorders of thyroid gland | E00-E07 | 1 |
| Diabetes | E10-E14 | 2 |
| Hemiplegia | G10-G14, G80-G83 | 2 |
| Malignancy | C00-C14, C15-C26, C27-C29, C30-C39, C40-C41, C42-C42, C43-C44, C45-C50, C51-C58, C59-C59, C60-C63, C64-C68, C69-C72, C73-C75, C81-C96, C97-C97 | 2 |
| Metastatic cancer | C76-C80 | 6 |
| Mild liver disease | B15, B16, B17, B18, B19, K70-K77 | 1 |
| Myocardial infarction | I20-I25 | 1 |
| Other chronic pulmonary disease | J41-J47, J60-J70 | 1 |
| Peripheral vascular disease | I70-I79, K55-K64 | 1 |
| Severe liver disease | I80-I89, I95-I99 | 3 |
| Ulcer | K20-K31 | 1 |

## Supplementary Figures

**Supplementary Figure S1.** Depiction of the results of the stratified subgroup analyses with adjusted risks of AUD-related hospitalizations in different sociodemographic subgroups that were diagnosed with an alcohol use disorder (ICD-10 codes: F10.0-F10.9) during the observational period (2009-2019) (CI = Confidence Interval, HR = Hazard Ratio).


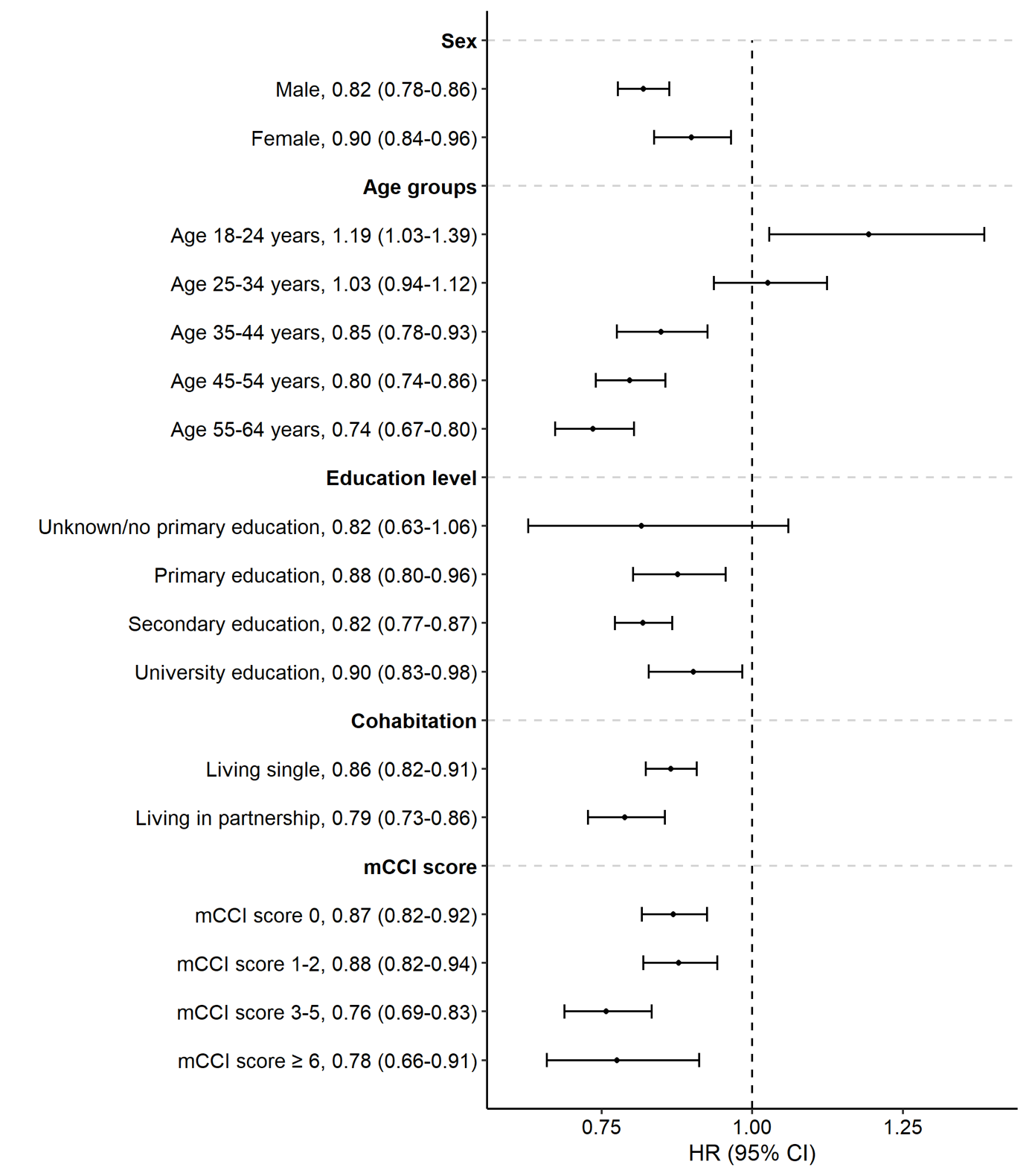

Supplement: Supplementary file 1 — Data S1. [file ACPS-152-94-s001.docx]
